# New protocol for rapid cassava multiplication in field conditions: a perspective on speed breeding

**DOI:** 10.3389/fpls.2023.1258101

**Published:** 2023-09-11

**Authors:** Leila Verena da Conceicão, Diego Fernando Marmolejo Cortes, Dominik Klauser, Michael Robinson, Eder Jorge de Oliveira

**Affiliations:** ^1^ Universidade Federal do Recôncavo da Bahia, Centro de Ciências Agrárias, Ambientais e Biológicas, Cruz das Almas, Bahia, Brazil; ^2^ Embrapa Mandioca e Fruticultura, Nugene, Cruz das Almas, Bahia, Brazil; ^3^ Syngenta Foundation for Sustainable Agriculture, Basel, Switzerland

**Keywords:** *Manihot esculenta* Crantz, selection, optimization, breeding cycle, seed treatment

## Abstract

Despite the economic and social importance, high-yielding cassava cultivars are only released after extensive research, mainly due to the low multiplication rate. This study aimed to assess the impact of using smaller-sized seed cuttings treated with agrochemicals (8MP) compared to the conventional planting size (16 cm) on genetic parameters, agronomic performance, and the ranking of cassava clones based on yield and growth attributes. The evaluation was carried out in clonal evaluation trial (CET), preliminary yield trial (PYT), and uniform yield trials (UYT). Additionally, a new selection scheme for cassava breeding programs was proposed. A total of 169 clones were evaluated, including 154 improved clones at different stages of selection and 15 local varieties used as checks. Field trials were conducted using both sizes of propagative material (8MP and 16 cm) in each phase of the breeding program. The data were analyzed using mixed models, considering the random effects of genotype and genotype-environment interaction (G×E) to determine variances and heritabilities. Bland-Altman concordance and correlation analysis of selection indices were employed to examine the consistency in the ranking of cassava clones using different seed cutting sizes. The distribution of variance components, heritabilities, means, and range of the 8MP and 16 cm trials in different phases of the cassava breeding program exhibited remarkable similarity, thereby enabling a comparative assessment of similar genetic effects. With a selection intensity of 30%, the concordance in clone ranking was 0.41, 0.57, and 0.85 in CET, PYT, and UYT trials, respectively, when comparing the selection based on 8MP and 16 cm trials. It is worth noting that the ranking of the top 15% remained largely unchanged. Based on the findings, proposed changes in the cassava selection scheme involve increasing the number of trials starting from the CET phase, early incorporation of G×E interaction, elimination of the PYT trial, reduction of the breeding cycle from 5 to 3 years, and a decrease in the time required for variety development from 11 to 9 years. These modifications are expected to lead to cost reduction and enhance the effectiveness of cassava breeding programs.

## 1 Introduction

Cassava (*Manihot esculenta* Crantz) is recognized as the second most significant source of starch globally (Stapleton, 2012). It serves as a fundamental crop for numerous agro-industrial processes worldwide. In addition to its industrial importance, cassava has been identified as a crucial crop for ensuring food security, particularly in sub-Saharan Africa (Ceballos et al., 2020; FAOSTAT, 2020). Despite the recent increase in global trade and exports of cassava products, leading to record levels of root production (Howeler et al., 2013), the average productivity of cassava in Brazil remains low, with 14.70 t.ha^-1^, significantly below the potential achieved by new cultivars (27.50 t.ha^-1^ in an annual cycle) (Oliveira et al., 2020).

Generally, the low root yield in Brazil can be attributed to various limiting factors, including poor soil fertility, inadequate propagation materials, low-yielding or unsuitable varieties for specific regions, and a lack of technological advancements in production systems (Andrade et al., 2019). Cassava breeding programs must address these factors to recommend high-yielding genotypes that meet the requirements of end-users. Considered the primary indicator of embraced technology by farmers across different technological levels, developing and adopting new cultivars remains a significant challenge for breeding programs. In both cases, the slow multiplication rate of cassava necessitates several years to develop new cultivars, and the adoption of these cultivars by farmers is often sluggish. This results in considerable frustrations, as the impacts of new cultivars are only perceived by farmers decades after their release.

Recent advancements in technologies such as genetic transformation, genomic selection, and gene editing, offer the potential for introgression or manipulation of genomic regions to confer adaptive and agronomic advantages (such as disease resistance, herbicide tolerance, and improved starch quality). However, these new approaches are more likely to succeed when integrated into breeding programs that also include conventional evaluation and adoption tests with farmers (Ceballos et al., 2020).

Conventional approaches used in cassava breeding typically rely on phenotypic recurrent selection, which leads to variation in the cassava selection cycle, usually ranging from 5 to 6 years. The overall time required to develop new cultivars is around 10 to 12 years (Oliveira et al., 2012; Wolfe et al., 2016; Ceballos et al., 2020). The main stages involved in phenotypic selection in cassava breeding programs include: i) crossing elite parents to produce progenies; ii) seedling evaluation tests (SET); iii) clonal evaluation tests (CET), where clones are planted without replication and selected based on traits with high heritability; iv) preliminary yield trials (PYT), where replicated evaluations begin and selection is based on traits with medium to high heritability; v) advanced yield trials (AYT), which involve larger plot sizes, multiple environments, and medium heritability traits; vi) uniform yield trials (UYT), characterized by evaluating clones in multiple replications, different environments, and multiple years to obtain more accurate data for traits with low heritability; vii) farmer trials, which utilize larger experimental plots and techniques adopted by collaborating farmers to assess the potential for new cultivar adoption and initiate multiplication stages; and viii) subsequent steps involve multiplication and release of new cultivars in target environments.

Due to the heterozygosity of cassava parents, the resulting progenies from crosses are genetically diverse, and each F_1_ plant is genetically distinct. According to Falconer and Mackay (1996), the genetic value of heterozygous genotypes is influenced by both additive and non-additive gene actions (dominance and epistasis). However, recurrent selection for parental development relies on genetic variability attributable to additive effects, as this component determines the long-term genetic gain of the population. On the other hand, clonal selection among F_1_ plants from different cassava progenies offers the advantage of fully exploiting both additive and non-additive effects (Bradshaw, 2016). Therefore, major cassava breeding programs have focused on population improvement (increasing additive value over time for various traits) and selection of clones for cultivar development (exploiting both additive and non-additive genetic effects).

The F_1_ progenies in cassava breeding are generated through sexual reproduction, but their multiplication is carried out through clonal propagation in subsequent selection stages. This propagation method presents one of the main challenges in cassava breeding programs, as it needs to balance phenotypic selection with the availability of propagation material. Cassava is traditionally propagated using stem cuttings, resulting in a low multiplication rate between mother and offspring plants, typically ranging from 1:5 to 1:10 (Oliveira et al., 2020). Therefore, new genotypes are initially tested as seedlings in non-replicated trials (SET). Selected clones from the SET trials are then clonally propagated to generate genetically identical plants, which are evaluated in non-replicated clonal trials (CET). Subsequently, the remaining genotypes undergo evaluation in larger experimental plots with multiple field replications (PYT). As the phenotypic selections progress, the number of genotypes is progressively reduced, and the selected ones are tested in larger experimental plots, multiple environments, and multiple years of cultivation (AYT and UYT).

This breeding scheme has seen only a few specific modifications in recent decades. Consequently, selection for less heritable traits has primarily been conducted in advanced stages such as AYT and UYT, where evaluations in different environments allow for the assessment of genotype × environment (G×E) interaction. G×E occurs when the contribution of alleles controlling a trait or their expression levels differ across environments (Gauch and Zobel, 1996). The selection of parents with high breeding values or high agronomic performance for the development of improved cultivars typically occurs 5 to 6 years after the initial crossings (following selection based on AYT and UYT tests).

There are several technologies available to increase the multiplication rate in cassava, such as *in vitro* micropropagation, seedling production from immature leaf buds, and the more recent hydroponic semi-autotrophic (SAH) method (IITA, 2017). However, these methods require specific infrastructure, such as greenhouses and specialized culture media/substrates, making them costlier for widespread use in cassava breeding. Additionally, these methods yield cassava seedlings as the final product rather than stem cuttings, which can create operational complications in the field and may not fully reflect the potential and production patterns of directly derived stem materials. On the other hand, Oliveira et al. (2020) recently developed a formulation composed of protective and growth stimulant agrochemicals, which has shown potential in maintaining germination rates and improving cassava resilience. According to the authors, 8 cm-long stem cuttings treated with the formulation (hereinafter referred to as 8MP) exhibited similar germination potential and agronomic performance to the local standard of cultivation using untreated 16cm cuttings (16 cm). Therefore, the main objectives of this study were: i) to evaluate the use of protective and growth stimulant agrochemicals in the genetic parameters related to yield traits and cassava growth at different phases of the cassava breeding program (CET, PYT, and UYT); ii) to assess the concordance in the ranking of cassava clones from the 8MP and 16cm treatments at different selection stages, considering agronomic traits and selection indices; iii) to evaluate the differences in agronomic performance between the 8MP and 16 cm treatments at different selection stages. Additionally, this manuscript discusses new perspectives to reduce the number of selective steps and thereby shorten the time required for the development of new cassava cultivars.

## 2 Material and methods

### 2.1 Plant material and experimental design

The cassava breeding program at Embrapa Mandioca e Fruticultura evaluated hybrids for the starch industry, as well as cassava varieties for fresh consumption commonly sold for the starch market during peak periods (**Table S1**). A total of 169 cassava clones were included in the evaluation, consisting of 154 new improved clones tested at different stages (CET, PYT, and UYT), and 15 local varieties (checks) used as agronomic standards in the target regions.

The CET utilized an augmented block design with 69 non-common treatment clones and 11 common controls, evenly distributed across 18 blocks (**Table 1**). Plots in the CET trial consisted of a single row with eight plants. For the PYT trials, a randomized complete block design with two replications was used. The number of new clones evaluated ranged from 19 to 71, depending on the availability of propagation material per trial. Additionally, between 11 and 13 controls were included. PYT experimental plots comprised two rows with eight plants each, resulting in a total of 16 plants per plot. The UYT tests were conducted using a randomized complete block design with three replications. Experimental plots consisted of 40 plants arranged in four rows with 10 plants each. In the UYT trials, 14 new clones were evaluated alongside 10 local controls.

**Table 1:** List of cassava clones evaluated for various agronomic traits at different breeding stages: clonal evaluation trial (CET), preliminary yield trial (PYT) and uniformed yield trial (UYT).

Breeding stage	New clones	Checks	N# total	Trial code	City/Location	Year	GPS
CET	69	11	80	BR.CET.21.PP1.16cm	Cruz das Almas (BA)/PP1	2022	12°39’14.6”S 39°04’47.1”W
	69	11	80	BR.CET.21.PP1.8MP	Cruz das Almas (BA)/PP1	2022	
PYT	19	12	31	BR.PYT.21.Candial.8MP	Cruz das Almas (BA)/Candial	2022	12°39’14.6”S 39°04’47.1”W
	67	11	78	BR.PYT.21.PP1.8MP	Cruz das Almas (BA)/PP1	2022	12°39’14.6”S 39°04’47.1”W
	71	13	84	BR.PYT.21.SJ.16cm	Laje (BA)/São Jorge	2022	13°06’29.4”S 39°18’34.3”W
	67	13	80	BR.PYT.21.SJ.8MP	Laje (BA)/São Jorge	2022	
UYT	14	10	24	BR.UYT.20.NH1.16cm	Laje (BA)/NH1	2021	13°06’38.8”S; 39°16’41.7”W
	14	10	24	BR.UYT.20.NH1.8MP	Laje (BA)/NH1	2021	
	14	10	24	BR.UYT.20.Roger.16cm	Laje (BA)/Roger	2021	13°09’04.5”S; 39°19’43.2”W
	14	10	24	BR.UYT.20.Roger.8MP	Laje (BA)/Roger	2021	
	14	10	24	BR.UYT.21.SJ.16cm	Laje (BA)/São Jorge	2022	13°07’36.7”S; 39°17’01.0”W
	14	10	24	BR.UYT.21.SJ.8MP	Laje (BA)/São Jorge	2022	
	14	10	24	BR.UYT.21.NH1.16cm	Laje (BA)/NH1	2022	13°07’36.7”S; 39°17’01.0”W
	13	10	23	BR.UYT.21.NH1.8MP	Laje (BA)/NH1	2022	

The cassava breeding program implemented the following trials:

- Two CET trials conducted in the 2022 harvest season in the city of Cruz das Almas (BA). One trial used conventional size planting material (referred to as “16cm”), while the other trial used reduced size material treated (8MP).
- Four PYT tests were conducted in the 2022 harvest season in the cities of Cruz das Almas (BA) and Laje (BA). One trial used the conventional 16cm size planting material, and three trials used the reduced size (8MP).
- Eight UYT tests were conducted in different production areas in the cities of Cruz das Almas (BA) and Laje (BA) in the 2021 and 2022 harvest seasons. Four trials used the conventional 16cm size planting material, and four trials used the reduced size material treated (8MP).

In each phase of the breeding program, field trials were set up using both sizes of propagation material to evaluate the agronomic performance of the cassava clones under the same cultivation environment. This allowed for the comparison of clone rankings under the same environmental influences. This was done for the CET trials planted in the same environment (tests BR.CET.21.PP1.16cm and BR.CET.21.PP1.8MP), PYT trials (tests BR.PYT.21.SJ.16cm and BR.PYT.21.SJ.8MP), and all UYT trials in the 2021 and 2022 growing seasons (**Table 1**). The other tests were used to verify the G×E interaction in early, intermediate and advanced stages of the breeding program.

The soil preparation process followed conventional methods, starting with the desiccation of weeds and subsequently plowing and harrowing twice to incorporate crop residues into the soil. A cassava planter was then utilized to create planting furrows and apply fertilizers based on the soil analysis of each specific area. Manual planting was carried out in the furrows using stakes measuring either 8 or 16 cm, depending on the treatment being applied. The cuttings used for planting were obtained from stems that were 11 to 12 months old, ensuring they were free from pests and diseases. These cuttings were placed horizontally along the planting line. The spacing between rows was set at 0.90 m, while the spacing between individual plants was maintained at 0.80 m. Following the planting process, post-planting cultural practices adhered to the nationally recommended guidelines for cassava, as outlined by Souza et al. (2006).

### 2.2 Cutting and treatment of seed cuttings

The cutting sizes utilized in the field trials were 8 cm and 16 cm, with the latter representing the commonly used propagation material size among Brazilian farmers. Typically, 8 cm cuttings contain 2 to 4 buds, while 16 cm cuttings can have 3 to 7 buds, depending on the clone. The cuttings were precisely prepared using an electric saw adjusted to the specific size required for each treatment. Following the cutting process, an absorption test was conducted on the 8 cm cuttings to help determine the appropriate pesticide dosage based on the volume of absorption prior to the treatment. Subsequently, the absorption volume per hectare (obtained by multiplying the number of cuttings per hectare by the absorption per cutting) was calculated. This information was used to estimate the total volume of product required (TVPR) using the formula): $TVPR=\;\frac{dosage\;of\;product*slurry\;required}{absorption\;volume*ha^{-1}}$ . In addition to the agrochemicals (thiamethoxam 21 g ha^-1^, mefenoxam 1.0 g ha^-1^, fludioxonil 1.3 g ha^-1^, thiabendazole 7.5 g ha^-1^), a binder (latex, 2%) was incorporated to enhance the adhesion of the treatment to the surface of the seed cuttings (Oliveira et al., 2020). All pesticides were applied using a water-based mixture to ensure uniform coverage of the cuttings. The pH value of the mixture was adjusted to a range of 6.5 to 7.0. Following treatment, the cuttings were allowed to dry for 8 hours at room temperature before being placed in polyethylene Raschel mesh bags, commonly used for storing onions and potatoes.

### 2.3 Traits assessed

Agronomic evaluations were conducted at the harvest time of the trials, which occurred 12 months after planting. The following traits were assessed:

#### 2.3.1 Plant height

- measured in meters, represents the vertical growth of cassava plants.

#### 2.3.2 Stand

- determined by counting the number of plants per plot and expressed as a percentage of the expected number of plants per plot. This indicates the establishment and population density of the plants.

#### 2.3.3 Number of stems per plant

- averaged from the evaluation of five plants in each plot, provides information about the prolificity habit and potential stem production.

#### 2.3.4 Plant architecture

- assessed on a scale of 1 to 5, indicating the overall size and branching pattern of the plants, where: 1: excellent – plants with no branches or with branches above 2.0 meters; 2: good – plants with branches above 1.60 meters or low branching, but at least 1.6 meters of erect stems; 3: medium – plants with branches over 1.20 meters or low branching, but at least 1.2 meters of erect stems; 4: poor – plants with branches over 0.80 meters or low branching, but with less than 0.80 meters of erect stems; and 5: very bad – highly branched clones with less than 0.80 meters of erect stems.

#### 2.3.5 Leaf retention

- assessed on a scale of 1 to 5, representing the coverage of leaves on the apical meristem: 1:<20% of apical meristem covered with leaves; 2: 20 - 39% of apical meristem covered with leaves; 3: 40 - 59% of apical meristem covered with leaves; 4: 60 - 79% of apical meristem covered with leaves; 5: >80% of apical meristem covered with leaves.

#### 2.3.6 Stem vigor

- evaluated on a scale of 1 to 5, indicating the thickness and strength of the stems: 1: very low vigor - stems<10 mm thick; 2: low vigor - stems between 11 and 15 mm thick; 3: medium vigor - stems between 16 and 20 mm thick; 4: high vigor - stems between 21 and 25 mm thick; 5: very high vigor - stems >25 mm thick.

#### 2.3.7 Dry matter content in the roots

- expressed as a percentage, was determined using the gravimetric method described by Kawano et al. (1987).

#### 2.3.8 Average number of roots per plant

- counted in a sample of five plants per plot, provides information about root system development.

#### 2.3.9 Shoot yield

assessed in t ha^-1^, represents the weight of stems, petioles, and leaves. This indicates the productivity of the above-ground biomass.

#### 2.3.10 Fresh root yield

- measured in t ha^-1^, represents the yield of fresh cassava roots.

#### 2.3.11 Dry root yield

- calculated by multiplying FRY by the DMC.

### 2.4 Data analysis

The CET tests were subjected to a variation analysis using the following model: $Y_{ij}=\mu +G_{i}+B_{j}+\varepsilon_{ij}$ , where $Y_{ij}$ is the observed value in the experimental plot of block *j* that received treatment *i* or the control treatment *i’* within block *j*. μ denotes the overall mean, $G_{i}$ represents the random effects of treatment *i* (where *i* = *i’* for the control treatment), $B_{j}$ is the fixed effect of block *j*, and ${\;}_{ij}$ is the random error associated with the portion of block *j* that received control treatment *i’* or regular treatment *i* within block *j*. The PYT and UYT tests were individually analyzed using a similar mixed model, but without considering the presence of a control treatment.

For the joint analysis of the PYT and UYT tests, a mixed model was employed: $Y_{ijk}=\mu +G_{i}+E_{k}+BE_{k}+GE_{ik}+\varepsilon_{ijk}$ , where $Y_{ijk}$ represents the observed value of the experimental plot in the *j* ^th^ block, of the *i* ^th^ genotype in the *k* ^th^ environment. μ denotes the overall mean, $G_{i}$ is the random effect of the *i* ^th^ genotype, $E_{k}$ represents the fixed effect of the *k* ^th^ environment, $BE_{jk}$ represents the fixed effect of the *j* ^th^ block on the *k* ^th^ environment, and ε ${\;}_{ijk}$ is the random error associated with the experimental plot in the *j* ^th^ block, of the *i* ^th^ genotype in the *k* ^th^ environment. All random effects are assumed to follow a normal and independent distribution.

Heritability estimates were obtained using two approaches: i) Heritability in the broad sense ( $H^{2}$ ), calculated using the equation $H^{2}=\frac{\sigma_{g}^{2}}{\sigma_{g}^{2}+\sigma_{i}^{2}\sigma_{e}^{2}}$ , where $\sigma_{\text{g}}^{2}$ represents the genotypic variance, $\sigma_{\text{i}}^{2}$ denotes the variance of the genotype by environment interaction, and $\sigma_{\text{e}}^{2}$ is the residual variance. ii) Cullis et al.’s (2006) heritability ( $H_{c}^{2}$ ), calculated using the equation: $H_{c}^{2}=1-\frac{\Delta BLUP}{2\sigma_{g}^{2}}$ , where Δ best linear unbiased prediction ( BLUP ) represents the average standard error of genotypic BLUPs. The data analysis using mixed models was performed in R software version 4.0.3 (R Core Team, 2020) with the lme4 package (Bates et al., 2015).

Boxplots, generated using the ggstatsplot package (Patil, 2021) in R software, were used to visualize the distribution and identify differences in agronomic traits across different trials in the cassava breeding program. Approximately 30% of cassava clones were selected in each trial and treatment to assess consistency in selection. The Mulamba and Mock (1978) selection index was used, which involves summing the ranks of each trait multiplied by predefined weights. The selection index (SI) was calculated as follows: SI=(PH×5)+(DMC×10)+(DRY×20)+(NHP×10)+(PlArc×−10)+(ShY×15)+(FRY×20)+(LeRet×10)+(Stand×15)+(StVig×10)+(NRP×2) . Here, each characteristic’s BLUP is multiplied by its respective economic weight. Genetic gain was calculated using the formula $G=h_{m}^{2}S$ , where G is the genetic gain and S is the BLUP deviation of the selected genotypes from the population mean, following Schmidt et al. (2019).

To assess the relationships and agreement between the different trials, Pearson correlations were calculated to examine the associations among the selection index values. Furthermore, the ranking of cassava clones was evaluated using the Bland and Altman graph (Bland & Altman, 1986). The Bland and Altman graph involved plotting the differences between each pair of clones (16cm - 8MP) on the vertical axis, while the average of the pair means [(16cm + 8MP)/2] was plotted on the horizontal axis. The 95% confidence limit, referred to as the Limits of Agreement (LOA), was determined as ±1.96 times the standard deviation (SD) of the bias.

## 3 Results

### 3.1 Analysis of variance components

**Figure 1:**
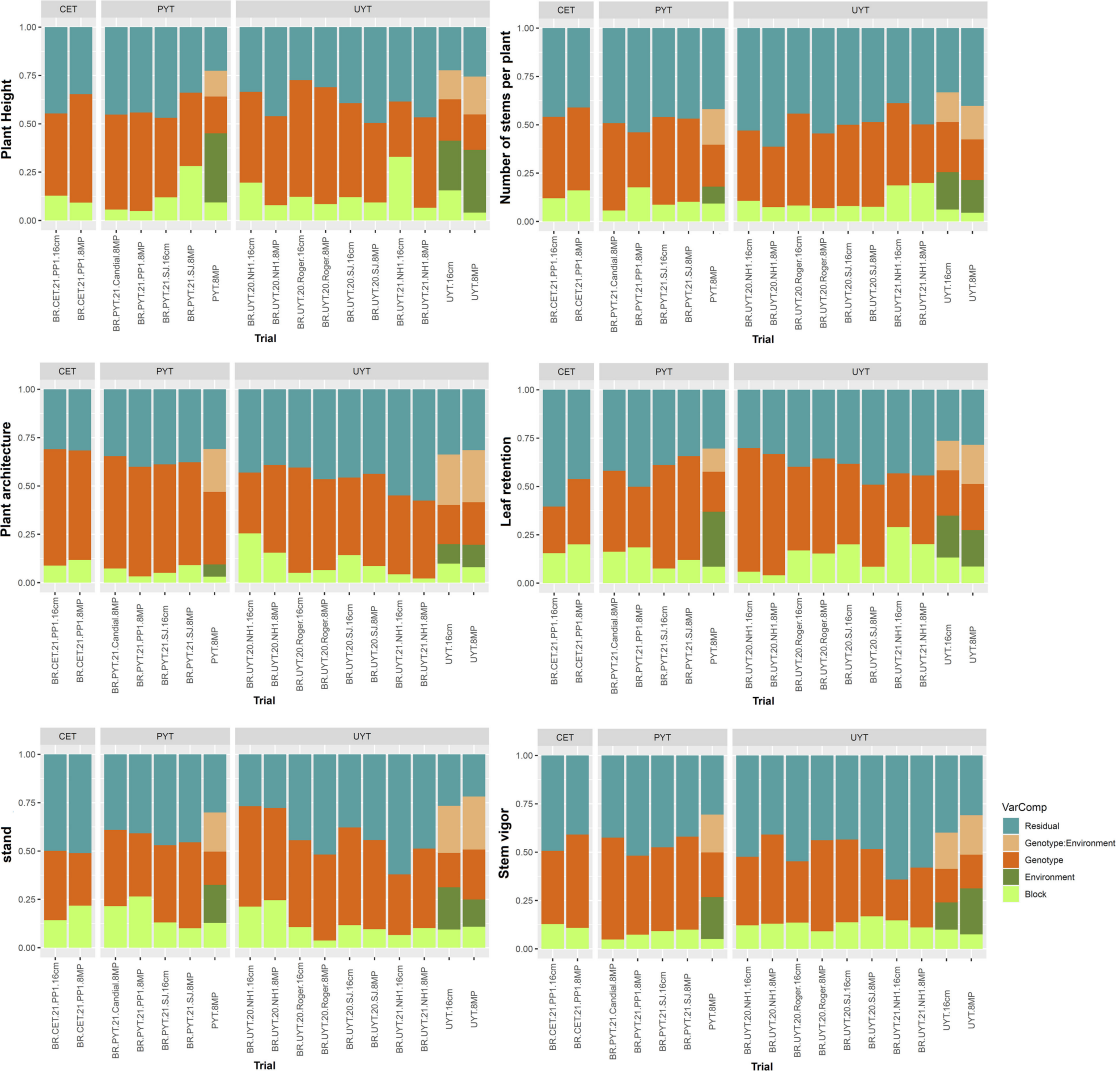
Phenotypic variance components estimates for traits related to plant growth and vigor (plant height, number of stems per plant, plant architecture, leaf retention, stand, and stem vigor) in the different trials of the cassava breeding program (clonal evaluation tests – CET, preliminary yield trial – PYT, and uniform yield trials – UYT, considering seed cuttings of standard size (16cm) and reduced size with treatment agrochemicals (8MP).The proportions of phenotypic variance attributed to each term were estimated using a mixed linear model and are presented in different colors.

Regarding the plant growth and vigor traits (PH, NHP, PlArc, LeRet, Stand, and StVig), the individual analysis of the different tests (CET, PYT, and UYT) showed that genetic and residual variances were predominant. In most cases, the genetic variance was higher, except for NHP and certain UYT (**Figure 1**). In the joint analysis of the three PYT 8MP trials, the environmental variance was higher for the PH trait, accounting for 35% of the total variance. For the PlArc trait, genetic variance played a more significant role, explaining 37% of the total variance. Similar patterns were observed in the joint analysis of the UYT 16cm and UYT 8MP trials, with the environmental variance being higher for the PH trait (25% and 32% of the total variance, respectively). The G×E interaction variance contributed relatively similarly to the genetic variance for most plant growth and vigor traits, accounting for 12% to 27% of the variation in G×E interaction and 17% to 37% of the variation in genetic variance.

**Figure 2:**
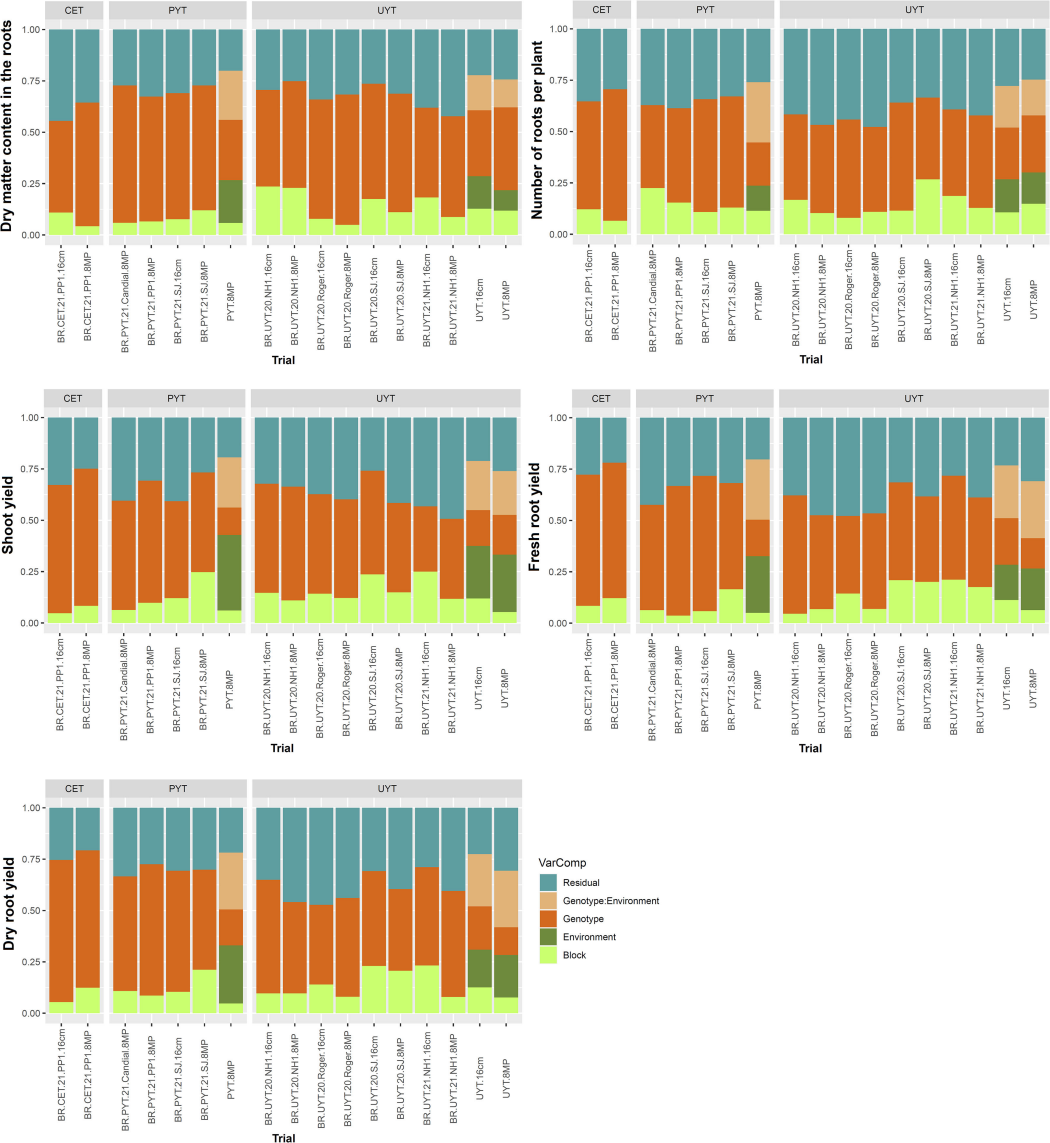
Phenotypic variance components estimates for yield traits (root dry matter content, number of roots per plant, shoot yield, fresh root yield, and dry root yield) in the different trials of the cassava breeding program (clonal evaluation tests – CET, preliminary yield trial – PYT and uniform yield trial – UYT, considering seed cuttings of standard size (16cm) and reduced size with agrochemical treatment (8MP) The proportions of phenotypic variance attributed to each term were estimated using a mixed linear model and are presented in different colors.

Regarding the yield traits (DMC, NRP, ShY, FRY, and DRY), the genetic variances in the individual analyses were greater than the variance due to the block and residual effects (**Figure 2**). However, in the joint analysis of the PYT and UYT tests, the environment had a greater influence on the ShY traits, accounting for 25% to 35% of the total variance. The G×E interaction variance was higher for the FRY and DRY characteristics, constituting approximately 27% of the total variance.

Based on **Figures 1** and **2**, the distribution of variance components in the 8MP and 16cm trials across different selection phases of the cassava breeding program (CET, PYT, and UYT), whether in individual or joint analysis, exhibited striking similarities. Therefore, in comparative terms, it is possible to capture similar genetic effects for the selection of the best clones and their advancement to subsequent evaluation stages.

### 3.2 Genetic parameters

In the individual CET trials, the heritability of plant growth and vigor traits were generally higher in the 8MP trial compared to the conventional 16cm trial, except for the Stand trait (**Table 2**). The LeRet, Stand, and StVig traits exhibited the lowest heritability in the 16cm test (BR.CET.21.PP1.16cm), with a range of 0.15 - 0.23 ( $H^{2}$ and $H_{c}^{2}$ ) for LeRet and 0.39 - 0.46 ( $H^{2}$ and $H_{c}^{2}$ ) for StVig. In the 8MP trial (BR.CET.21.PP1.8MP), these heritability values were higher but still moderate, ranging from 0.24 to 0.27 ( $H^{2}$ and $H_{c}^{2}$ ) for Stand and 0.53 to 0.57 ( $H^{2}$ and $H_{c}^{2}$ ) for StVig. On the other hand, the yield traits in the CET tests showed high heritability values, ranging from 0.75 to 0.76 ( $H^{2}$ and $H_{c}^{2}$ ) for DMC and 0.82 to 0.93 ( $H^{2}$ and $H_{c}^{2}$ ) for DRY. The differences in heritability values between the 8MP and 16cm trials for the yield traits were smaller compared to the plant growth and vigor traits.

**Table 2:** Broad-sense heritability ( $H^{2}$ ) and Cullis heritability ( $H_{c}^{2}$ ) for growth, vigor and root yield traits in different cassava breeding trials (clonal evaluation trials – CET, preliminary yield trial - PYT and uniform yield trial - UYT), considering standard cuttings size (16cm) and small cutting size with agrochemical treatment (8MP).

Level	Trial	PH		NSP		PlArc		LeRet		Stand		StVig		DMC		NRP		ShY		FRY		DRY	
		$H^{2}$	$H_{c}^{2}$	$H^{2}$	$H_{c}^{2}$	$H^{2}$	$H_{c}^{2}$	$H^{2}$	$H_{c}^{2}$	$H^{2}$	$H_{c}^{2}$	$H^{2}$	$H_{c}^{2}$	$H^{2}$	$H_{c}^{2}$	$H^{2}$	$H_{c}^{2}$	$H^{2}$	$H_{c}^{2}$	$H^{2}$	$H_{c}^{2}$	$H^{2}$	$H_{c}^{2}$
Individual	BR.CET.21.PP1.16cm	0.46	0.51	0.41	0.46	0.79	0.81	0.15	0.23	0.33	0.40	0.39	0.46	0.47	0.54	0.69	0.73	0.79	0.81	0.83	0.86	0.82	0.85
	BR.CET.21.PP1.8MP	0.77	0.76	0.68	0.65	0.76	0.79	0.40	0.41	0.24	0.27	0.53	0.57	0.75	0.76	0.82	0.81	0.88	0.89	0.91	0.91	0.93	0.93
Individual	BR.PYT.21.SJ.16cm	0.69	0.76	0.42	0.57	0.75	0.83	0.68	0.79	0.44	0.60	0.58	0.70	0.86	0.87	0.81	0.89	0.76	0.82	0.89	0.93	0.87	0.87
	BR.PYT.21.SJ.8MP	0.59	0.65	0.39	0.49	0.69	0.80	0.74	0.84	0.49	0.65	0.59	0.72	0.85	0.85	0.81	0.87	0.81	0.87	0.73	0.84	0.72	0.76
	BR.PYT.21.Candial.8MP	0.67	0.75	0.47	0.63	0.74	0.84	0.49	0.65	0.62	0.74	0.66	0.76	0.81	0.88	0.54	0.70	0.67	0.74	0.68	0.76	0.69	0.77
	BR.PYT.21.PP1.8MP	0.60	0.67	0.22	0.32	0.67	0.76	0.29	0.42	0.48	0.59	0.43	0.54	0.78	0.83	0.58	0.70	0.76	0.81	0.79	0.86	0.79	0.82
Joint	PYT.8MP	0.35	0.60	0.18	0.42	0.49	0.75	0.30	0.62	0.48	0.59	0.30	0.58	0.47	0.67	0.22	0.44	0.18	0.36	0.21	0.40	0.23	0.40
Individual	BR.UYT.20.NH1.16cm	0.79	0.84	0.33	0.54	0.37	0.59	0.81	0.91	0.79	0.90	0.11	0.25	0.75	0.86	0.50	0.72	0.80	0.86	0.72	0.85	0.73	0.85
	BR.UYT.20.NH1.8MP	0.50	0.72	0.21	0.42	0.65	0.82	0.77	0.89	0.75	0.87	0.56	0.75	0.82	0.90	0.46	0.69	0.68	0.82	0.48	0.70	0.49	0.71
	BR.UYT.20.Roger.16cm	0.83	0.91	0.58	0.77	0.65	0.82	0.54	0.76	0.52	0.73	0.25	0.49	0.75	0.87	0.54	0.75	0.63	0.81	0.53	0.74	0.40	0.64
	BR.UYT.20.Roger.8MP	0.73	0.86	0.37	0.62	0.58	0.79	0.68	0.82	0.45	0.66	0.54	0.75	0.80	0.90	0.43	0.67	0.59	0.78	0.50	0.72	0.55	0.75
	BR.UYT.21.SJ.16cm	0.61	0.82	0.45	0.69	0.45	0.70	0.54	0.75	0.66	0.83	0.49	0.73	0.82	0.93	0.70	0.86	0.80	0.92	0.72	0.86	0.72	0.84
	BR.UYT.21.SJ.8MP	0.39	0.62	0.44	0.69	0.54	0.78	0.53	0.73	0.51	0.73	0.41	0.64	0.78	0.91	0.60	0.80	0.54	0.76	0.56	0.77	0.52	0.72
	BR.UYT.21.NH1.16cm	0.51	0.69	0.56	0.79	0.37	0.62	0.29	0.54	0.26	0.48	0.11	0.26	0.59	0.80	0.54	0.77	0.36	0.56	0.76	0.91	0.74	0.89
	BR.UYT.21.NH1.8MP	0.54	0.68	0.29	0.47	0.33	0.53	0.63	0.78	0.42	0.65	0.18	0.33	0.58	0.78	0.49	0.71	0.36	0.57	0.55	0.76	0.52	0.74
Joint	UYT.16cm	0.38	0.76	0.34	0.79	0.18	0.58	0.36	0.78	0.45	0.66	0.13	0.55	0.55	0.88	0.34	0.77	0.22	0.59	0.28	0.68	0.26	0.65
Joint	UYT.8MP	0.23	0.64	0.19	0.65	0.22	0.63	0.35	0.75	0.45	0.66	0.19	0.59	0.67	0.93	0.35	0.79	0.25	0.66	0.21	0.42	0.19	0.48

PH, plant height; NHP, number of stems per plant; PlArc, plant architecture; LeRet, leaf retention; Stand, plot stand; StVig, stem vigor; DMC, dry matter content in roots; NRP, average number of roots per plant; ShY, shoot yield; FRY, fresh root yield; and DRY, dry root yield.

In the case of individual PYT tests, most of the traits showed similar median heritability compared to the CET, with slightly higher heritability values in the PYT tests, except PlArc trait (**Table 2**). However, agronomic traits exhibited higher heritability, similar to the CET tests. For a direct comparison, the trials BR.PYT.21.SJ.16cm and BR.PYT.21.SJ.8MP, which were planted side by side in the same environment, showed very similar $H^{2}$ and $H_{c}^{2}$ values for plant growth, vigor, and yield traits. For instance, $H^{2}$ values ranged from 0.73 to 0.89 for FRY and 0.69 to 0.75 for PlArc, while $H_{c}^{2}$ values ranged from 0.84 to 0.93 for FRY and 0.80 to 0.83 for PlArc. Regarding the other PYT 8MP trials conducted in different environments, the heritability values of BR.PYT.21.Candial.8MP were similar to those of BR.PYT.21.SJ.16cm and BR.PYT.21.SJ.8MP trials. However, the BR.PYT.21.PP1.8MP trial showed slightly lower heritability, particularly for traits such as NHP, LeRet, and StVig. Furthermore, while the individual variance analysis of the tests revealed medium to high heritabilities, the joint analysis of the three PYT 8MP tests resulted in low heritability value for most traits, except for PlArc, where the influence of environmental variance and G×E interaction was of relatively low magnitude.

The heritability values of the individual UYT were generally comparable to those of the CET and PYT, although some variations were observed. Notably, traits such as plant height (PH), leaf retention (LeRet), and stand (Stand) exhibited higher heritability in certain field trials (**Table 2**). The StVig trait displayed the widest range of heritability estimates, with values ranging from 0.11 to 0.56 for $H^{2}$ and 0.25 to 0.75 for $H_{c}^{2}$ . Similarly, the ShY trait showed moderate to high heritability, with $H^{2}\;$ ranging from 0.36 to 0.80 and $H_{c}^{2}\;$ ranging from 0.56 to 0.92. Among all traits, dry matter content (DMC) consistently displayed the highest heritability across the UYT trials, aligning with the findings from the PYT trials.

Comparing the heritability between the UYT trials with 16cm and 8MP treatments planted in the same environment (BR.UYT.20.NH1.16cm and BR.UYT.20.NH1.8MP, BR.UYT.20.Roger.16cm and BR.UYT.20.Roger.8MP, BR.UYT.20.SJ.16cm and BR.UYT.20.SJ.8MP, BR.UYT.21.NH1.16cm and BR.UYT.21.NH1.8MP), minor differences were observed in the $H^{2}$ and $H_{c}^{2}$ values, except for a few specific cases. For instance, there were slight variations in StVig heritability between the BR.UYT.20.NH1, BR.UYT.20.Roger, and BR.UYT.21.NH1 trials, as well as differences in LeRet heritability in the BR.UYT.21.NH1 trials and PlArc heritability in the BR.UYT.20.NH1 trials. Nevertheless, overall, the heritability estimates of the joint analysis of the UYT 16cm and UYT 8MP trials were similar for all traits, except for DMC, LeRet, and NHP. These findings indicate the potential of incorporating this approach into breeding programs, as it may expedite the development of new cassava varieties by reducing the required time.

### 3.3 Distribution of growth, vigor and productivity characteristics of cassava clones

**Figure 3:**
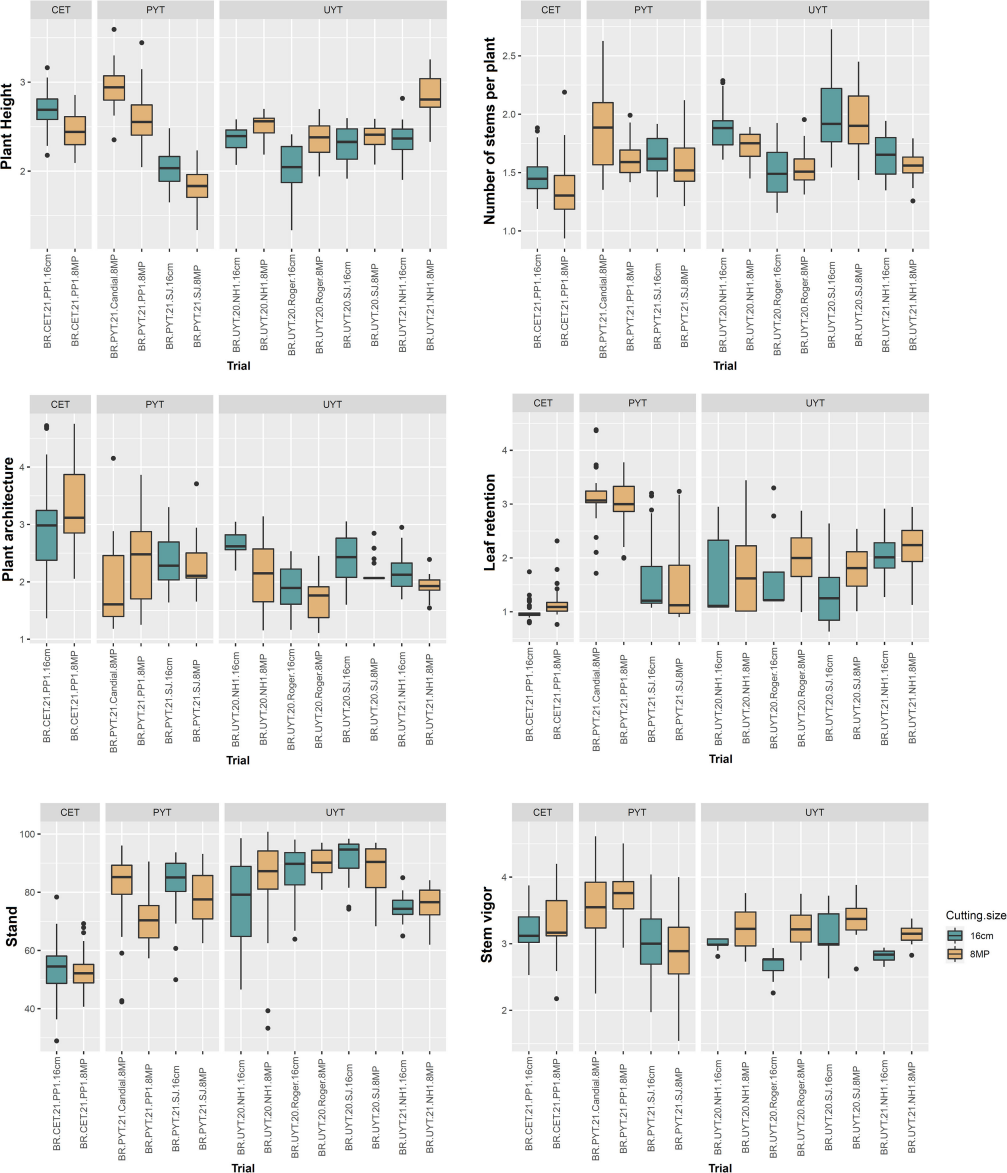
Box plot of best linear unbiased prediction + the overall mean (BLUP.U) on different cassava genotypes evaluated for plant growth and vigor traits (plant height – PH, number of stems per plant – NHP, plant architecture – PlArc, leaf retention – LeRet, stand – Stand, stem vigor – StVig) in the different tests of the cassava breeding program (clonal evaluation trial – CET, preliminary yield trial – PYT and uniform yield trial – UYT, considering seed cuttings of standard size (16cm) and reduced size with agrochemical treatment (8MP).

**Figure 4:**
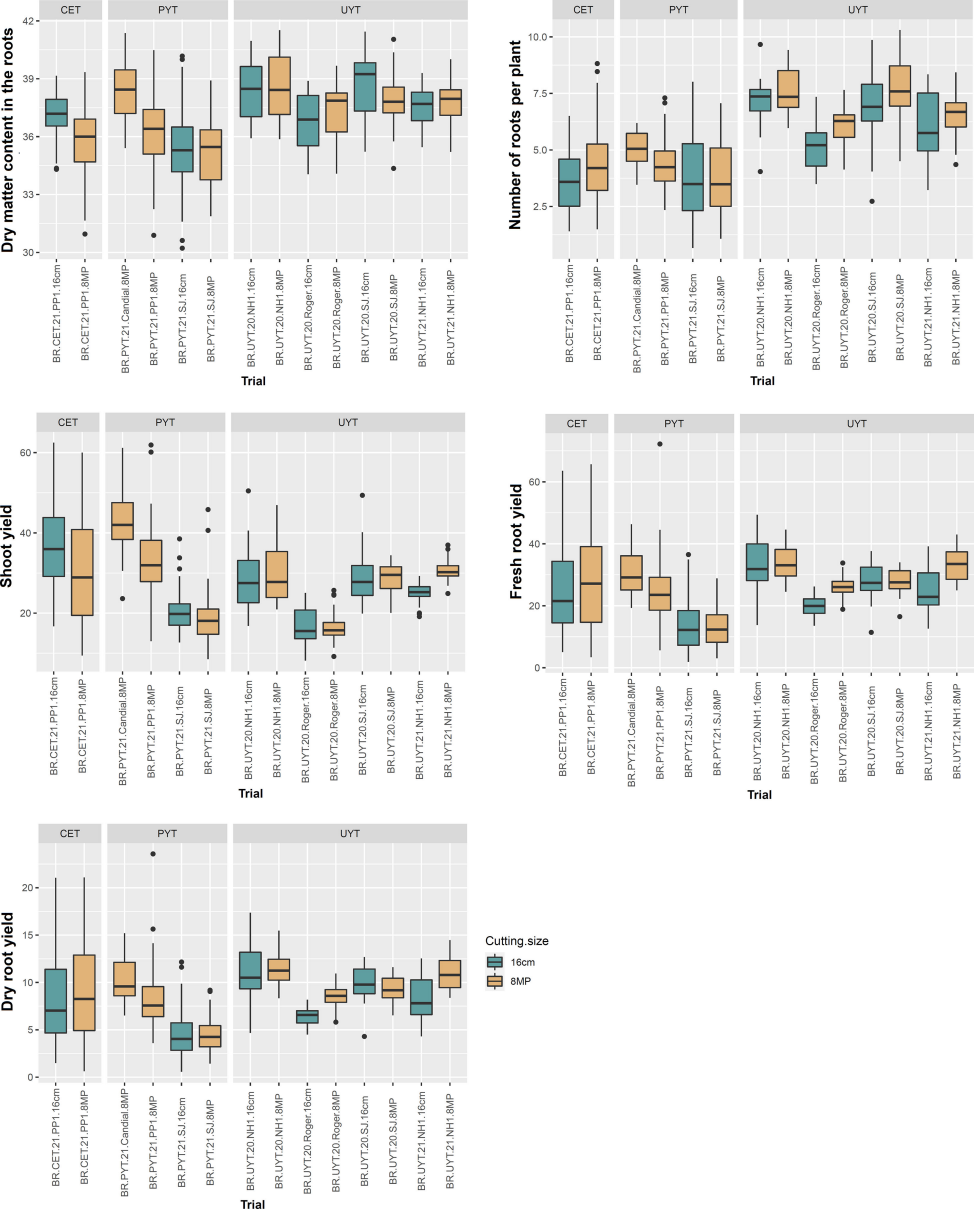
Box plot of best linear unbiased prediction + the overall mean (BLUP.U) on different cassava genotypes evaluated for yield traits (dry matter content in roots - DMC, average number of roots per plant - NRP, above-ground yield - ShY, fresh root yield - FRY, and dry root yield - DRY) in the different tests of the cassava breeding program (clonal evaluation trial – CET, preliminary yield trial – PYT and uniform yield trial – UYT, considering seed cuttings of standard size (16cm) and reduced size with agrochemical treatment (8MP).

**Figures 3** and **4** display boxplots illustrating the best linear unbiased predictions (BLUPs) combined with the general average of the tests (referred to as BLUP.U) in the cassava breeding program. Among the different trials, the PYT trials exhibited the highest variation in BLUP.U for most of the plant growth and vigor traits (PH, PlArc, Stand, and StVig) compared to the other trials. Conversely, the UYT trials demonstrated the greatest variation in BLUP.U for LeRet, while the CET trials showed the highest variation for PlArc (**Figure 3**). Regarding the productive traits, the CET, PYT, and UYT tests displayed the highest variation in BLUP.U, in that order, with the exception of DMC (**Figure 4**).

In general, the distribution of BLUP.U for trials with different treatments (16cm × 8MP) planted side by side in the same experimental area exhibited remarkable similarity across various evaluated traits. This finding aligns with the results obtained from the analysis of variance components and heritability estimation for the measured traits. On the other hand, the two additional PYT 8MP trials (BR.PYT.21.Candial.8MP and BR.PYT.21.PP1.8MP), conducted in environments distinct from BR.PYT.21.SJ.16cm and BR.PYT.21.SJ.8MP, displayed significant variations in agronomic performance for nearly all growth, vigor, and yield traits (**Figures 3**, **4**). These differences could potentially be attributed to G×E, as these trials were conducted in contrasting experimental areas with varying pathogen pressures and soil conditions.

### 3.4 Coincidence in the ranking of cassava clones in the different selection stages

The coincidence analysis of cassava clones aimed to compare the performance of cassava clones under different treatments, specifically standard cuttings (16cm) and reduced cuttings size with agrochemical treatment (8MP). The analysis focused on the ranking of clones based on various selection criteria, such as plant growth and vigor traits, in the CET, PYT, and UYT. In the CET tests, a correlation analysis between the selection indices (SI) of the 16cm and 8MP treatments revealed a moderate correlation (R = 0.41***), indicating some agreement in the ranking of clones. Out of the 82 clones evaluated, 13 clones (53%) were selected in both treatments, while 12 clones were selected in either one of the treatments, and the remaining clones were not selected in either treatment. The Bland-Altman analysis further confirmed the agreement between the two treatments, showing that the top-ranked clones based on SI were consistently selected in both the 16cm and 8MP treatments. Additionally, clones ranked between the 14^th^ and 42^nd^ positions were selected by either treatment, while clones above the last ranking were not selected by either treatment.

In the PYT, the comparison was made between the SI of the 16cm test and the BLUP.U from the joint analysis of the three 8MP trials. The correlation analysis indicated a medium magnitude correlation (R = 0.57***), suggesting a better agreement in clone selection between these two types of trials. Out of the selected clones, 12 clones (48%) were chosen in both the 16cm and 8MP trials, while 13 clones were selected in either one of the treatments, and the remaining clones were not selected. The Bland-Altman analysis supported the agreement between the two treatments, with the top-ranked clones consistently selected in both the 16cm and 8MP trials. Moreover, clones ranked from the 13^th^ to the 40^th^ positions were selected by one or more treatments, indicating some variability in clone selection between the different trials. Notably, the PYT 8MP trials selected five additional clones beyond the 40^th^ position, which may be attributed to the evaluation of these clones in multiple environments.

**Figure 5:**
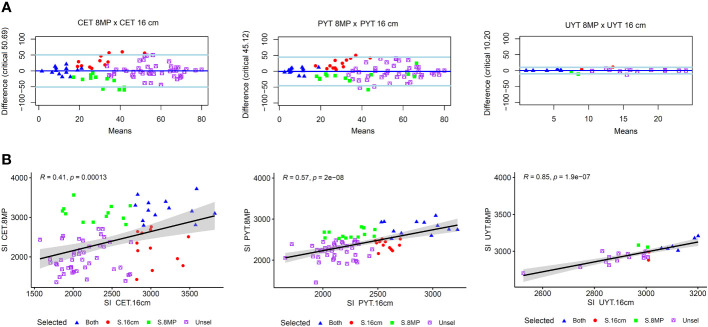
Concordance in the ranking of cassava clones **(A)** and the Pearson’s correlation between the selection indices **(B)** obtained in various trials of the cassava breeding program. These trials include clonal evaluation trials (CET), preliminary yield trials (PYT), and uniform yield trials (UYT). The analysis takes into account seed cuttings of standard size (16cm) and reduced size with agrochemical treatment (8MP). The graphs represent the selection indexes derived from the BLUP.U of the joint analysis, considering evaluations in different environments and years, such as the PYT 8MP and UYT 16cm and 8MP tests. In the correlation graphs, the black line represents the linear regression curve, and the gray band represents the 95% confidence interval. The Bland-Altman analysis assesses the agreement in clone ranking, where the solid dark blue line represents the estimated bias, and the two light blue lines depict the upper and lower confidence limits at 95% agreement.

In the UYT trials, the cassava clones were also compared based on the BLUP.U obtained from the combined analysis of the 16cm and 8MP treatments. Unlike the CET and PYT tests, there was a strong correlation between the selection indices (SI) of these two sets of trials (R = 0.85***), indicating consistent selection patterns (**Figure 5**). The selection rates showed little dispersion, with a high agreement between the clones selected in both the 16cm and 8MP trials. Out of the 24 clones evaluated in the CET trials, 7 clones were selected (at a selection intensity of 30%), and among them, 6 clones (83%) were selected in both the 16cm and 8MP trials, while only 2 clones were exclusively selected in either one of the treatments. The Bland-Altman analysis demonstrated a smaller difference in the ranking of cassava clones between the PYT 16cm and 8MP trials compared to the CET and UYT trials (**Figure 5**). However, consistent with the other tests, the top-ranked clones in the standard 16cm trials also performed well in the 8MP treatments. Only four clones that ranked between 8^th^ and 10^th^ in either the UYT 16cm or UYT 8MP trials were selected, with two clones from each treatment.

### 3.5 Use of the 8MP approach in the selection scheme in cassava breeding

**Figure 6:**
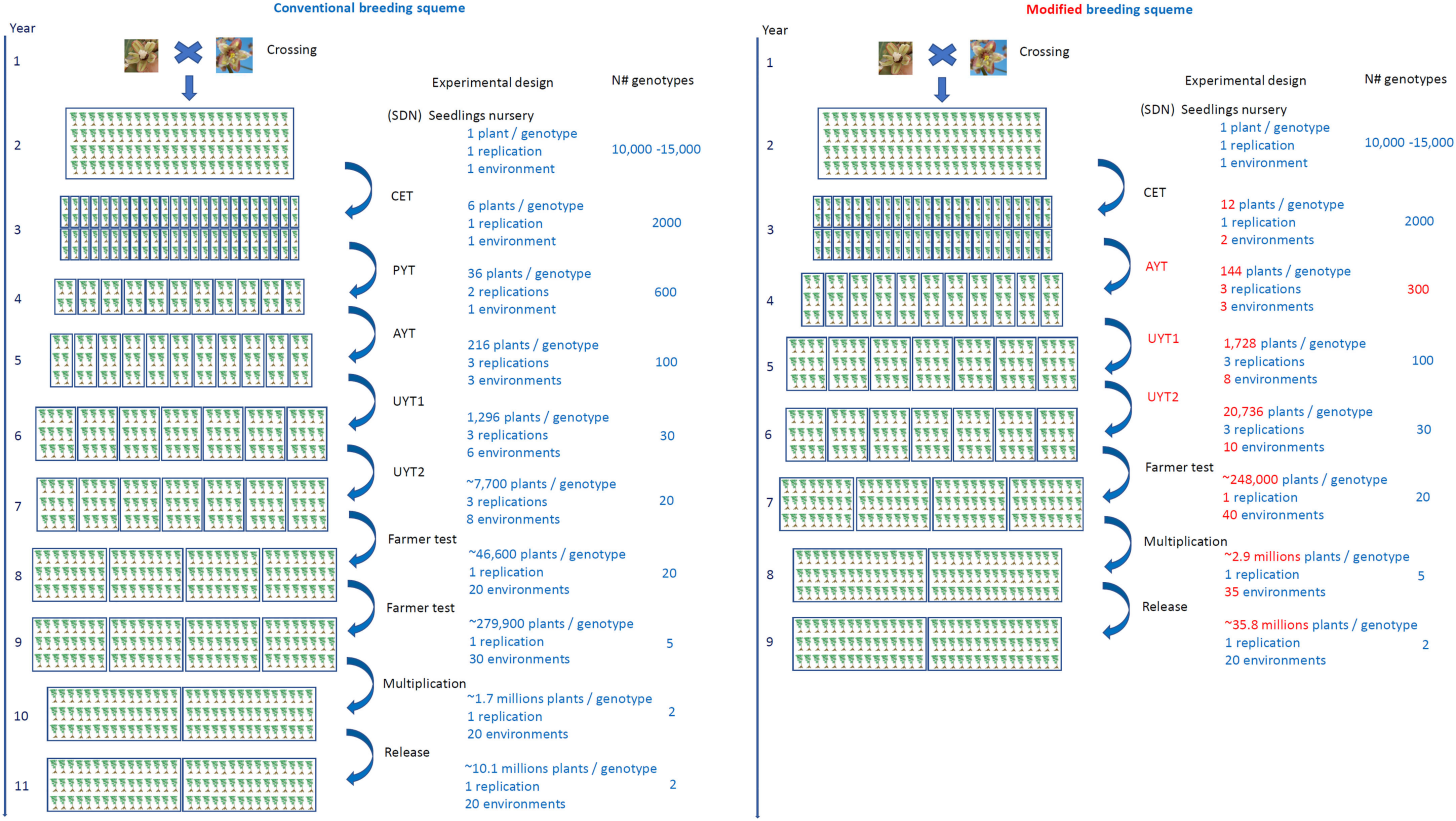
Conventional cassava breeding scheme (left) using 16 cm cuttings and alternative scheme using 8 cm long seed cuttings treated with agrochemicals (right). The scheme represents the main stages of the cassava genetic breeding program, which are SDN, seedlings nursey; CET, clonal evaluation trial; PYT, Preliminary yield trial; AYT, Advanced yield trial; UYT, Uniformed yield trial; Farmer test; Multiplication; Release. In the alternative scheme (right), the main modifications compared to the conventional system are highlighted in red.

In order to expedite the development of new cassava cultivars, the utilization of agrochemicals treated cassava seedlings with reduced size (8MP) at various stages of the breeding program, comparable to the system and commercial standard of propagation material (16 cm), can yield significant benefits. This approach holds promise for reducing the time required to develop new cassava cultivars. To capitalize on the advantages of the 8MP strategy, we propose a revised breeding scheme that incorporates its use starting from the initial clonal selection (CET) stage. By implementing this scheme, we aim to analyze the potential impact of the 8MP approach on expediting cassava development (**Figure 6**).

Under the conventional cassava breeding scheme, the number of plants within a plot, the number of repetitions within a trial, and the number of environments and years of cultivation analyzed in each step depend on the quantity of propagation material generated in the preceding selective phase. This quantity is closely tied to the cassava propagation rate, which typically ranges from 1:5 to 1:10 for most cassava clones (Oliveira et al., 2020). For comparative purposes, we consider an average multiplication ratio of 1:6, which is highly realistic for the majority of cassava clones. Typically, the selection process involves one SET, one CET, one PYT, three AYT, approximately 14 UYT tests conducted over two years of cultivation (to generate essential information on cultivation and use value - VCU - for registration and variety protection). Subsequently, farmer tests are conducted to agronomically validate the clones across a minimum of 40 environments, with at least 2000 plants per location. Once VCU data is obtained and validated with end users of the new cultivars, the multiplication stage of the new cultivars commences in collaboration with licensed nurseries, lasting for another year. The final stage involves the release of the variety. Consequently, this entire process typically spans at least 11 years, considering the time required for crossing and generating the segregating progenies (**Figure 6**).

When implementing the 8MP approach in CET, the first significant change is conducting the trials in two environments instead of just one (**Figure 6**). This modification allows for the exploration of G×E interactions at an early stage in the breeding program. It not only aids in the selection process but also increases the production of propagation material. Once the CET is harvested and the best clones are selected, the second important change is the elimination of PYT tests since a sufficient amount of propagation material is already available for assembling AYT tests with three replications and three environments. The UYT tests, conducted in the first and second years, would proceed as usual, serving the purpose of generating phenotypic information for registration and protection of cassava cultivars. The only difference in the UYT tests would be the larger number of plants available for each clone, allowing for evaluations in 18 environments instead of the conventional 14 (**Figure 6** and **Table 3**).

**Table 3:** Comparison of conventional multiplication and selection schemes (untreated seedlings 16cm – 16cm) and 8 cm long treated with agrochemicals and alternative (8MP) of cassava breeding programs.

Breeding stage	Conventional cassava breeding squeme (16cm)								
	N# total manivas		N# manivas/rep	Rep	Env	N# genotypes		Total Area (ha)	N# manivas/genotype
SET	1		1	1	1	10.000	15.000	1	1
CET	6		6	1	1	2.000		1	6
PYT	36		18	2	1	600		2	36
AYT	216		24	3	3	100		2	216
UYT	1.296		72	3	6	30		3	1.296
UYT	7.776		324	3	8	20		11	7.776
Farmer test	46.656		2.333	1	20	20		67	46.656
Farmer test	279.936		9.331	1	30	5		101	279.936
Multiplication	1.679.616		83.981	1	20	2		242	1.679.616
Release	10.077.696		503.885	1	20	2		1.451	10.077.696
Breeding stage	Alternative breeding squeme using 8MP approach								
SET	1	1		1	1	10.000	15.000	1	1
CET	12	6		1	2	2.000		2	12
AYT	144	16		3	3	300		3	144
UYT	1.728	72		3	8	100		12	1.728
UYT	20.736	691		3	10	30		45	20.736
Farmer test	248.832	6.221		1	40	20		358	248.832
Multiplication	2.985.984	85.314		1	35	5		1.075	2.985.984
Release	35.831.808	1.791.590		1	20	2		5.160	35.831.808

SDN, nursey seedlings; CET, clonal evaluation trial; PYT, Preliminary yield trial; AYT, Advanced yield trial; UYT, Uniformed yield trial; Farmer test; Multiplication; Release. In the alternative scheme (right), the main modifications compared to the conventional system are highlighted in red.

The farm test stage involves conducting agronomic validation tests in a minimum of 40 environments, with a minimum of 2000 plants, which is typically achieved by carrying out two evaluation cycles in the conventional system. However, with the 8MP approach, it would be possible to reduce the farm test validation period by one year. After one year following the UYT tests, more than 6000 plants of each clone would be available to set up the 40 farm tests. In the multiplication stage for farmers, another change arises as it becomes possible to work with over 35 registered multiplying agents instead of the 20 in the conventional procedure. Additionally, the 8MP approach allows for the production of over 2.9 million plants, compared to the 1.7 million in the conventional strategy. This increased production capacity significantly impacts the diffusion of new cassava varieties (**Figure 6** and **Table 3**).

The schematic layout (**Figure 6**) has been carefully designed to align with Brazilian regulations governing the protection and registration of novel cassava cultivars. This comprehensive framework has been tailored to fulfill the specific mandate of conducting a minimum of two UYT trials, ensuring compliance with the rigorous criteria for assessing the VCU of these new cultivars. This encompasses considerations such as the stipulated minimum number of plants per trial and the imperative inclusion of diverse testing environments. However, is worth considering alternative, more streamlined approaches that can potentially expedite the process, such as: Year 1. crossing, Year 2. seedling nursery, Year 3. clonal evaluation trial, Year 4. advanced yield trial, multi-locational, Year 5. uniform yield trial, multi-locational, Year 6. on-farm testing and validation, Year 7. formal variety release. Although this alternative plan requires validation, our compiled data strongly suggests a high likelihood of realizing this elevated level of optimization within the cassava breeding program.

## 4 Discussion

### 4.1 Variance components and genetic parameters in trials with standard and reduced cuttings size

Breeding programs aim to develop superior genotypes with desirable traits that provide competitive advantages over existing varieties. The success of selecting these superior genotypes relies on the presence of genetic variability and understanding the environmental influence on trait expression. The genetic potential of breeding populations can be assessed by estimating genetic and phenotypic parameters, enabling the selection of favorable traits. Traits with higher genetic variance allow for early selection, independent of environmental effects. However, despite cassava’s wide adaptability to various environmental conditions, most varieties exhibit limited adaptability and show significant G×E interaction effects (Tumuhimbise et al., 2014; Bakare et al., 2022). This is evident in the cultivation of more than 80 local and improved cassava varieties across Brazil, spanning from the north to the south.

For most agronomic traits evaluated, environmental effects and G×E interactions predominated, except for traits such as PlArc and DMC, where genetic variances played a more prominent role, particularly in multi-environment trials. Previous studies have also reported a lesser influence of the environment and G×E interaction on these traits (Okeke et al., 2018; Rabbi et al., 2022). Interestingly, the variances observed in the 8MP (reduced size) and 16cm (standard size) trials were quite similar across different trials, indicating that genetic effects were captured similarly and that there was a strong association between phenotypic and genotypic values of similar magnitude.

Among the genetic parameters evaluated, heritability is considered an important index as it quantifies the proportion of phenotypic variation that is attributed to genetic differences among individuals (Falconer and Mackay, 1996). In general, the $H^{2}$ and $H_{c}^{2}$ values were higher in trials with replications, such as PYT and UYT, for most traits. This can be attributed to the increased precision of the estimates due to the inclusion of replications and the evaluation of a larger number of diverse environments in the UYT trials. A study by Freitas et al. (2018) comparing genetic parameters and genetic gains in early stages of cassava breeding using full-sib (F_1_) and self-pollinated (S_1_) families also found higher heritability estimates for traits like dry matter content, fresh and dry root yield in replicated trials (PYT) compared to non-replicated trials in a single environment (CET). The authors emphasized the low agreement in clone selection between the CET and PYT trials and recommended increasing the number of clones per family and using moderate selection intensity, particularly in the CET, to discard poorly performing clones in the early stages of selection.

Although the heritability increased with improved experimental designs in the PYT and UYT trials, the differences in $H^{2}\;$ and $H_{c}^{2}\;$ parameters between the 16cm and 8MP treatments were generally low, especially in the joint analysis of each phase of the breeding program. This indicates that both treatments captured similar proportions of the genetic variance in the data. Therefore, it is important to implement trials with field replications and evaluations in diverse environments, even in the initial stages of cassava breeding programs, to obtain more accurate estimates of genetic parameters, which are often used in the selection of clones for advancement. According to Grüneberg et al. (2009), increasing the number of trials (locations and years) is crucial to enhance the heritability of traits, and incorporating G×E interaction in the prediction of breeding values at an early stage increases the potential for genetic gain per unit of time.

### 4.2 Ranking ability of clones in trials with standard and reduced cuttings size

The progress of cassava clones in different stages of the breeding program (CET, PYT, AYT, and UYT) relies heavily on the quality of experimental trials and the accuracy of phenotyping traits under selection. This is particularly true for traits with low heritability such as fresh root yield, starch content, and disease resistance (Diniz and Oliveira, 2019). Typically, these traits are evaluated in advanced selection stages with replicated trials conducted across multiple environments.

After phenotyping, cassava breeders rank the genotypes using selection indices that consider economic weights assigned to various traits under analysis. This enables decision-making regarding the advancement or elimination of genotypes in subsequent selection stages. In the present study, the ranking of cassava clones using both the conventional approach (16cm) and reduced propagation material size (8MP) showed promising results. The rankings varied from moderate magnitudes in CET (R = 0.41***) and PYT (R = 0.57***) to high in UYT trials (R = 0.85***). In practical terms, this means that with a selection intensity of 30% of clones, more than half of them were selected using both approaches (16cm and 8MP) in the CET and PYT trials. In the UYT, over 80% of clones were selected using both approaches. Moreover, when employing a lower selection intensity (<30%), the agreement in the ranking of cassava clones would be even higher, as the top clones were consistently selected by both approaches.

While increasing the number of trials in the early stages of cassava breeding programs has the potential to improve heritability estimates, considering the G×E interaction can complicate selection by altering genotype rankings across different growing environments. However, the modeling of phenotypic stability and identification of the most stable genotypes have become crucial in delivering successful cassava cultivars (Jiwuba et al., 2020; Bakare et al., 2022). Evaluating the G×E interaction is essential in designing an optimal breeding strategy for developing genotypes that adapt well to target environments. Introducing this component into breeding schemes at an earlier stage allows for faster selection of parental controls for new breeding cycles and, most importantly, reduces the time required for developing new cassava varieties.

### 4.3 New selection scheme based on the reduced cutting size (8MP) to accelerate the development of cassava cultivars

Both the commercial production system and the conventional planting system in breeding programs rely on clonal multiplication through cassava stem cuttings, which are directly planted in the field. However, the commercial system often uses low-quality planting materials that are frequently contaminated with pests and diseases, leading to compromised crop germination, establishment, and profitability. In breeding programs, the quality of cuttings is maintained to ensure the identification of the maximum genetic potential of new genotypes. Nonetheless, both systems are limited by the propagation rate of cassava, typically ranging from 1:5 to 1:10, depending on the variety (Oliveira et al., 2020). Consequently, producing sufficient planting materials on a large scale takes several years.

Various techniques for rapid cassava propagation have been described in the literature. *In vitro* micropropagation is one such technique, capable of producing high-quality planting material on a large scale and within a short period (Aladele and Kuta, 2008; Feyisa, 2023). However, the main drawback of *in vitro* micropropagation is the high cost of seedling production, primarily due to the sophisticated laboratory requirements and post-bottle management procedures that users often find challenging (Legg et al., 2022). As a result, this methodology is generally limited to producing basic materials for other lower-cost rapid propagation techniques.

Another technique involves using shorter planting materials, such as mini-cuttings measuring 5 cm in length and containing 2 - 3 buds, which are planted in growth chambers. This method has shown high efficiency in the rapid multiplication and preservation of local varieties threatened by climate-related issues, such as severe droughts in certain regions of Brazil. Recently, Neves et al. (2020) improved a rapid multiplication technique based on immature leaf buds, which can achieve an annual multiplication rate of 1:72. Additionally, the semi-autotrophic hydroponic (SAH) method, which leverages the plant’s autotrophic capacity to grow in environments with improved gas exchange and more natural conditions (Rigato et al., 2000), has been adapted for rapid cassava propagation. However, all the aforementioned methodologies primarily focus on producing plantlets requiring hardening as the final product, which poses a challenge for their practical adoption. End users, such as producers, cooperatives, and agroindustries, often lack the necessary expertise to plant cassava plantlets, particularly on a large scale. Furthermore, planting plantlets incurs additional costs, mainly due to increased labor requirements for planting and maintaining plants in the field, as well as the need for specialized herbicide management.

Recently, Oliveira et al. (2020) developed a strategy for rapid multiplication of cassava in the field using agrochemicals that protect and stimulate growth, known as protective and growth stimulant agrochemicals. This technique enabled the reduction of cassava propagation material size to 8 cm without compromising germination and crop establishment, while maintaining yields comparable to conventional untreated cassava propagation material (16 cm). Although this technology shows great promise for increasing the multiplication rate in cassava seed production systems, its application in breeding programs is currently limited. However, the findings of our study demonstrate that the use of the 8MP approach in CET tests would enable the inclusion of tests in different environments during the initial phase of the breeding program. This allows for the evaluation of clone performance and the G×E at an earlier stage, as compared to the current breeding scheme where G×E interaction evaluation typically occurs during AYT tests, and in some cases, PYT tests. Thus, a significant change in this new breeding scheme would involve the elimination of PYT trials, while AYT trials would be conducted with a reduced number of plants but with the same number of replications and evaluation environments as conventional methods. Both methods would incorporate UYT tests over two consecutive years to generate the minimum required information for the registration and protection of cassava cultivars. However, with the 8MP multiplication scheme, it would be possible to evaluate the clones in at least 8 environments in the first year and 10 environments in the second year, whereas the conventional system typically includes the evaluation of clones in approximately 6 and 8 environments during the first and second year of UYT trials, respectively. This enhanced evaluation approach allows for a more comprehensive assessment of clone performance under diverse environmental conditions.

In the conventional system, two years of evaluation in farm test trials are implemented, resulting in the generation of approximately 1.7 million seed cuttings. In contrast, the 8MP cutting multiplication system introduces a significant change by implementing only one year of evaluation in farm test trials across about 40 different cultivation environments. This approach would yield a substantially greater number of cuttings-seeds (~2.9 million) for the subsequent multiplication step, intended for distribution to end-users. The introduction of the 8MP approach and the modification of the breeding scheme proposed in our study represent important advancements in cassava multiplication and improvement. These changes have the potential to enhance the evaluation of clones in various environments at an earlier stage, expedite the breeding cycle, and increase the availability of high-quality planting material for farmers. By integrating these innovations, we can accelerate the development of improved cassava varieties, ultimately benefiting both breeders and end-users.

In recent years, cassava breeders have shown increased interest in exploring alternative selection methods. Ceballos et al. (2007) proposed a series of changes to the improvement scheme adopted by CIAT, which shares similarities with the alterations proposed in our study. These changes primarily focused on the initial stages of selection and included eliminating a multiplication stage after seedling evaluation (F1C1), implementing CET tests with 8 plants instead of the conventional 6, and conducting PYT tests with three replicates instead of a single field replicate. By adopting this new selection scheme, the time required to reach the tests was reduced from 66 to 58 months after botanical seed germination. In our study, the 8MP approach, when integrated with the cassava breeding program, could potentially lead to an even more significant reduction in the time required to reach AYT trials (36 months). This approach could also impact the overall time needed for cultivar development, potentially reducing it to 9 years compared to the conventional procedure of 11 years. Furthermore, it would have implications for the final quantity of propagation material available for distribution to farmers.

While the focus of our results presentation has primarily been on the ranking and behavior of clones as they progress through the breeding program, the proposed changes would introduce important shifts in cassava population improvement. Traditionally, improvement has been based on clone performance rather than individual genetic values. Only recently have changes been made to this paradigm through the implementation of genomic selection in various cassava breeding programs (Oliveira et al., 2012; Wolfe et al., 2016; Wolfe et al., 2017; Andrade et al., 2019; Torres et al., 2019). Genomic selection involves selecting parents based on genomic estimated breeding values (GEBV) predicted at the seedling stage, without incorporating phenotypic data from the current generation. While this strategy reduces the cassava breeding cycle to 24 months (including the crossing stages), complex traits with a significant influence of non-additive effects, such as fresh root yield, dry root yield, and resistance to diseases like cassava brown streak disease (CBSD), exhibit low predictive accuracy (Wolfe et al., 2017; Andrade et al., 2022; Ozimati et al., 2022). This is likely due to the fact that genomic prediction models have not been updated with phenotypic data from current generations. However, with the new selection scheme proposed in our study, the true genetic values of cassava clones, along with their general combining ability, could be obtained through the AYT trials, which would last up to 36 months, including the crossing stages. This would enable genomic prediction to be performed after obtaining AYT data, allowing for the updating of prediction models and improving predictive accuracy with the inclusion of one more year in the improvement cycle.

### 4.4 Final remarks

The urgency of addressing climate change and its impact on global food production necessitates the development of more sustainable solutions. Cassava, being a resilient and versatile crop, holds great potential as a food, feed, and industrial crop in the face of predicted adverse climate scenarios. However, the genetic improvement of cassava has been slower compared to other crops like cereals, primarily due to its long reproductive cycle and low multiplication rate. Therefore, there is a need to innovate traditional breeding schemes to expedite the availability of improved varieties that exhibit resistance to diseases and stresses associated with rising temperatures and water scarcity, while also meeting the quality requirements of end-users.

In this study, we propose a novel breeding scheme that enables the early implementation of multi-environment tests in breeding programs using smaller seed cuttings treated with a formulation containing protective agrochemicals and growth stimulants. This approach allows for the estimation of genetic parameters and the ranking of cassava clones in a manner similar to conventional selection schemes. However, the significant advantage of our approach is the incorporation G×E interaction evaluation at the CET, eliminating the need for separate multiplication and selection steps. Furthermore, it increases the availability of propagation material for cultivars to be distributed to end-users in the final stages of the breeding program and reduces the time required to develop new cultivars from 11 to 9 years. This reduction in time also translates to cost savings in genetic programs.

By implementing this new scheme, we aim to accelerate the genetic improvement of cassava, enabling the development of more productive varieties with enhanced resistance to diseases and stresses associated with climate change. These innovations will contribute to ensuring food security and meeting the demands of end-users while addressing the challenges posed by a changing climate.

## Data availability statement

The original contributions presented in the study are included in the article/**Supplementary Material**. Further inquiries can be directed to the corresponding author.

## Author contributions

LC: Formal Analysis, Investigation, Methodology, Validation, Visualization, Writing – original draft. DC: Investigation, Methodology, Software, Supervision, Validation, Writing – review & editing. DK: Conceptualization, Project administration, Resources, Writing – review & editing. MR: Conceptualization, Resources, Supervision, Writing – review & editing. EO: Conceptualization, Funding acquisition, Methodology, Project administration, Supervision, Writing – review & editing.
