# Supplementary material for: New protocol for rapid cassava multiplication in field conditions: a perspective on speed breeding
Source: Front Plant Sci. 2023 Sep 11;14:1258101. doi: 10.3389/fpls.2023.1258101 (PMC10518405; doi:10.3389/fpls.2023.1258101)
Supplement: Supplementary file 1 [file Table_1.docx]

**Table S1**. List of cassava clones evaluated for various agronomic traits at different breeding stages: clonal yield trial (CET), preliminary yield trial (PYT) and uniformed yield trial (UYT), with its root characteristics.

| Clone | Breeding Stage | | | Pulp color | Cortex color | External color of roots | Type |
| --- | --- | --- | --- | --- | --- | --- | --- |
| BR-11-34-41 | - | - | UYT | White | White or cream | Light brown | Sweet |
| BR-11-34-45 | CET | PYT | - | White | White or cream | Light brown | Bitter |
| BR-11-34-64 | CET | PYT | UYT | White | White or cream | Light brown | Intermedium |
| BR-14-001-24 | - | - | UYT | White | White or cream | Light brown | Intermedium |
| BR-14-006-02 | - | - | UYT | White | White or cream | Light brown | Sweet |
| BR-14-006-32 | - | - | UYT | White | White or cream | Light brown | Sweet |
| BR-14-010-11 | - | - | UYT | White | White or cream | White or cream | Intermedium |
| BR-14-025-42 | - | - | UYT | White | White or cream | White or cream | Sweet |
| BR-14S1-Eucalipto-34 | - | - | UYT | White | White or cream | Light brown | Intermedium |
| BR-17-006-12 | - | - | UYT | Cream | Pink | Light brown | Bitter |
| BR-17-006-62 | - | - | UYT | White | White or cream | Light brown | Intermedium |
| BR-17-012-16 | - | - | UYT | White | White or cream | Light brown | Bitter |
| BR-17-012-19 | - | - | UYT | White | White or cream | Light brown | Bitter |
| BR-17-012-59 | - | - | UYT | Cream | Yellow | Light brown | Bitter |
| BR-17-013-05 | - | - | UYT | Cream | Yellow | Light brown | Bitter |
| BR-17-020-10 | - | - | UYT | White | White or cream | White or cream | Sweet |
| BR-17S1-030-71 | - | - | UYT | White | White or cream | White or cream | Sweet |
| BR-18GS-111-90 | - | PYT | - | White | White or cream | Light brown | Bitter |
| BR-19-002-14 | - | PYT | - | Cream | White or cream | Light brown | Sweet |
| BR-19-002-181 | - | PYT | - | Cream | White or cream | Light brown | Sweet |
| BR-19-002-196 | - | PYT | - | Cream | White or cream | Light brown | Sweet |
| BR-19-002-216 | - | PYT | - | Cream | White or cream | Light brown | Bitter |
| BR-19-002-235 | - | PYT | - | Cream | White or cream | Light brown | Bitter |
| BR-19-002-252 | - | PYT | - | White | White or cream | Light brown | Intermedium |
| BR-19-002-254 | - | PYT | - | Cream | Yellow | Light brown | Intermedium |
| BR-19-002-386 | - | PYT | - | White | White or cream | Light brown | Bitter |
| BR-19-002-406 | - | PYT | - | Cream | White or cream | Light brown | Intermedium |
| BR-19-002-408 | - | PYT | - | Yellow | White or cream | Light brown | Intermedium |
| BR-19-002-434 | - | PYT | - | Cream | White or cream | Light brown | Intermedium |
| BR-19-002-513 | - | PYT | - | Cream | White or cream | Light brown | Sweet |
| BR-19-002-59 | - | PYT | - | Yellow | White or cream | Light brown | Sweet |
| BR-19-004-7 | - | PYT | - | White | Pink | Light brown | Bitter |
| BR-19-013-19 | - | PYT | - | White | White or cream | White or cream | Intermedium |
| BR-19-016-10 | - | PYT | - | White | White or cream | White or cream | Bitter |
| BR-19-016-12 | - | PYT | - | White | White or cream | White or cream | Bitter |
| BR-19-016-17 | - | PYT | - | White | White or cream | White or cream | Intermedium |
| BR-19-016-21 | - | PYT | - | White | White or cream | White or cream | Bitter |
| BR-19-016-23 | - | PYT | - | White | White or cream | White or cream | Bitter |
| BR-19-016-27 | - | PYT | - | White | White or cream | Light brown | Bitter |
| BR-19-018-10 | - | PYT | - | White | White or cream | White or cream | Bitter |
| BR-19-018-15 | - | PYT | - | White | White or cream | White or cream | Intermedium |
| BR-19-018-23 | - | PYT | - | White | White or cream | Yellow | Bitter |
| BR-19-018-7 | - | PYT | - | White | White or cream | Yellow | Bitter |
| BR-19-018-9 | - | PYT | - | White | White or cream | Yellow | Intermedium |
| BR-19F1wx-020-12 | - | PYT | - | White | White or cream | White or cream | Bitter |
| BR-19F1wx-054-29 | - | PYT | - | White | Yellow | Yellow | Bitter |
| BR-19F2Polwx-025-11 | - | PYT | - | White | Yellow | Yellow | Bitter |
| BR-19F2Polwx-042-24 | - | PYT | - | White | White or cream | Light brown | Sweet |
| BR-19F2Polwx-053-3 | - | PYT | - | White | White or cream | Light brown | Intermedium |
| BR-19F2Polwx-053-31 | - | PYT | - | White | White or cream | White or cream | Bitter |
| BR-19F2Polwx-053-58 | - | PYT | - | White | White or cream | White or cream | Intermedium |
| BR-19F2Polwx-053-64 | - | PYT | - | White | White or cream | Light brown | Sweet |
| BR-19F2Polwx-053-9 | - | PYT | - | White | Pink | Light brown | Bitter |
| BR-19F2Polwx-054-16 | - | PYT | - | White | White or cream | White or cream | Bitter |
| BR-19F2wx-045-8 | - | PYT | - | White | White or cream | Light brown | Bitter |
| BR-19F2wx-155-25 | - | PYT | - | Yellow | Yellow | Light brown | Bitter |
| BR-19F2wx-161-1 | - | PYT | - | Cream | White or cream | Light brown | Bitter |
| BR-19F2wx-204-15 | - | PYT | - | White | White or cream | Light brown | Intermedium |
| BR-19F2wx-215-3 | - | PYT | - | White | White or cream | Light brown | Bitter |
| BR-19F2wx-223-4 | - | PYT | - | White | Yellow | Light brown | Bitter |
| BR-19F2wx-241-24 | - | PYT | - | White | White or cream | Light brown | Bitter |
| BR-19F2wx-241-35 | - | PYT | - | White | Pink | Light brown | Bitter |
| BR-19F2wx-241-5 | - | PYT | - | White | White or cream | Light brown | Bitter |
| BR-19F2wx-252-1 | - | PYT | - | White | White or cream | Light brown | Bitter |
| BR-19F2wx-255-4 | - | PYT | - | Yellow | Yellow | Light brown | Bitter |
| BR-19F2wx-255-7 | - | PYT | - | Cream | White or cream | Light brown | Intermedium |
| BR-19F2wx-257-2 | - | PYT | - | White | White or cream | Yellow | Bitter |
| BR-19F2wx-281-2 | - | PYT | - | White | White or cream | Light brown | Intermedium |
| BR-19F2wx-282-1 | - | PYT | - | White | White or cream | Light brown | Intermedium |
| BR-19F2wx-287-1 | - | PYT | - | White | White or cream | Light brown | Bitter |
| BR-19F2wx-299-14 | - | PYT | - | Cream | White or cream | Light brown | Bitter |
| BR-19F2wx-338-1 | - | PYT | - | White | Yellow | Light brown | Bitter |
| BR-19F2wx-341-1 | - | PYT | - | White | Pink | Light brown | Bitter |
| BR-19F2wx-348-21 | - | PYT | - | White | White or cream | Light brown | Bitter |
| BR-19F2wx-348-3 | - | PYT | - | White | White or cream | Light brown | Bitter |
| BR-19F2wx-348-8 | - | PYT | - | White | White or cream | Light brown | Bitter |
| BR-19F2wx-356-2 | - | PYT | - | White | White or cream | Light brown | Bitter |
| BR-19F2wx-367-6 | - | PYT | - | Yellow | White or cream | Yellow | Bitter |
| BR-19F2wx-371-36 | - | PYT | - | White | White or cream | Light brown | Intermedium |
| BR-19F2wx-372-4 | - | PYT | - | White | White or cream | Light brown | Bitter |
| BR-19F2wx-376-26 | - | PYT | - | White | White or cream | White or cream | Sweet |
| BR-19F2wx-376-30 | - | PYT | - | White | White or cream | Yellow | Bitter |
| BR-19F2wx-381-4 | - | PYT | - | White | White or cream | Light brown | Bitter |
| BR-19F2wx-385-69 | - | PYT | - | Yellow | White or cream | Light brown | Bitter |
| BR-19F2wx-394-2 | - | PYT | - | White | White or cream | Yellow | Intermedium |
| BR-19F2wx-404-60 | - | PYT | - | White | White or cream | Light brown | Bitter |
| BR-19F2wx-405-7 | - | PYT | - | White | White or cream | Light brown | Bitter |
| BR-19F2wx-420-1 | - | PYT | - | White | Yellow | Light brown | Bitter |
| BR-20-007-01 | CET | - | - | White | Yellow | Light brown | Bitter |
| BR-20-007-10 | CET | - | - | White | White or cream | Yellow | Bitter |
| BR-20-015-05 | CET | - | - | White | White or cream | White or cream | Bitter |
| BR-20-015-06 | CET | - | - | White | White or cream | Yellow | Sweet |
| BR-20-015-07 | CET | - | - | White | White or cream | White or cream | Bitter |
| BR-20-037-01 | CET | - | - | Yellow | White or cream | Light brown | Sweet |
| BR-20-047-04 | CET | - | - | White | White or cream | Light brown | Bitter |
| BR-20-047-06 | CET | - | - | White | White or cream | Light brown | Bitter |
| BR-20-047-17 | CET | - | - | White | White or cream | Yellow | Bitter |
| BR-20-047-34 | CET | - | - | White | White or cream | Yellow | Bitter |
| BR-20-047-38 | CET | - | - | White | White or cream | Yellow | Intermedium |
| BR-20-050-04 | CET | - | - | White | White or cream | Light brown | Bitter |
| BR-20-050-15 | CET | - | - | White | White or cream | Yellow | Bitter |
| BR-20-050-16 | CET | - | - | White | White or cream | Light brown | Bitter |
| BR-20-051-06 | CET | - | - | White | White or cream | Yellow | Bitter |
| BR-20-069-06 | CET | - | - | White | White or cream | Yellow | Bitter |
| BR-20-069-23 | CET | - | - | White | Yellow | Light brown | Sweet |
| BR-20-069-28 | CET | - | - | White | White or cream | Yellow | Bitter |
| BR-20-069-30 | CET | - | - | White | White or cream | Light brown | Sweet |
| BR-20-069-31 | CET | - | - | White | White or cream | White or cream | Sweet |
| BR-20-069-34 | CET | - | - | White | White or cream | Light brown | Bitter |
| BR-20-069-45 | CET | - | - | White | White or cream | Yellow | Bitter |
| BR-20-070-03 | CET | - | - | Yellow | White or cream | Yellow | Sweet |
| BR-20-073-02 | CET | - | - | White | White or cream | Yellow | Bitter |
| BR-20-073-07 | CET | - | - | White | White or cream | Light brown | Bitter |
| BR-20-073-17 | CET | - | - | White | White or cream | Yellow | Sweet |
| BR-20-074-05 | CET | - | - | White | White or cream | Yellow | Bitter |
| BR-20-074-07 | CET | - | - | White | White or cream | White or cream | Bitter |
| BR-20-074-08 | CET | - | - | White | White or cream | Yellow | Bitter |
| BR-20-074-16 | CET | - | - | White | White or cream | Yellow | Bitter |
| BR-20-074-17 | CET | - | - | White | White or cream | Light brown | Bitter |
| BR-20-074-22 | CET | - | - | White | White or cream | Yellow | Intermedium |
| BR-20-084-06 | CET | - | - | White | White or cream | Light brown | Sweet |
| BR-20-084-07 | CET | - | - | White | White or cream | Light brown | Bitter |
| BR-20-086-04 | CET | - | - | White | White or cream | Light brown | Bitter |
| BR-20-094-12 | CET | - | - | White | White or cream | Yellow | Intermedium |
| BR-20-094-15 | CET | - | - | White | White or cream | Light brown | Bitter |
| BR-20-094-18 | CET | - | - | White | White or cream | Light brown | Bitter |
| BR-20-094-20 | CET | - | - | White | White or cream | Light brown | Bitter |
| BR-20-094-24 | CET | - | - | White | White or cream | Light brown | Bitter |
| BR-20-094-81 | CET | - | - | White | White or cream | Light brown | Intermedium |
| BR-20-094-93 | CET | - | - | White | White or cream | Light brown | Sweet |
| BR-20-095-03 | CET | - | - | White | White or cream | Light brown | Bitter |
| BR-20-096-02 | CET | - | - | White | White or cream | Light brown | Sweet |
| BR-20-098-03 | CET | - | - | White | White or cream | Light brown | Bitter |
| BR-20-105-02 | CET | - | - | White | White or cream | Light brown | Sweet |
| BR-20-106-02 | CET | - | - | White | White or cream | Yellow | Intermedium |
| BR-20-106-07 | CET | - | - | White | White or cream | Yellow | Bitter |
| BR-20-106-14 | CET | - | - | White | White or cream | Light brown | Sweet |
| BR-20F1wx-005-01 | CET | - | - | White | White or cream | White or cream | Intermedium |
| BR-20F2Polwx-004-08 | CET | - | - | White | White or cream | Light brown | Intermedium |
| BR-20F2Polwx-008-09 | CET | - | - | White | White or cream | Light brown | Bitter |
| BR-20F2Polwx-012-01 | CET | - | - | White | White or cream | Yellow | Sweet |
| BR-20F2Polwx-014-01 | CET | - | - | White | White or cream | Light brown | Bitter |
| BR-20F2Polwx-017-06 | CET | - | - | White | White or cream | Light brown | Intermedium |
| BR-20F2Polwx-025-105 | CET | - | - | White | White or cream | Light brown | Bitter |
| BR-20F2Polwx-025-24 | CET | - | - | White | White or cream | Light brown | Bitter |
| BR-20F2wx-004-03 | CET | - | - | White | Pink | Light brown | Bitter |
| BR-20F2wx-010-01 | CET | - | - | White | White or cream | Light brown | Bitter |
| BR-20F2wx-032-03 | CET | - | - | White | Pink | Light brown | Bitter |
| BR-20F2wx-042-01 | CET | - | - | White | White or cream | Light brown | Bitter |
| BR-20F2wx-050-01 | CET | - | - | White | White or cream | Light brown | Sweet |
| BR-20F2wx-066-03 | CET | - | - | White | White or cream | White or cream | Intermedium |
| BR-20F2wx-072-01 | CET | - | - | White | White or cream | Light brown | Sweet |
| BR-20S1-006-02 | CET | - | - | Cream | Yellow | Yellow | Bitter |
| BR-20S2-007-01 | CET | - | - | White | White or cream | White or cream | Bitter |
| BR-20S2-007-02 | CET | - | - | White | White or cream | Light brown | Bitter |
| BR-20S2-010-01 | CET | - | - | White | White or cream | Light brown | Bitter |
| BR-20S2-012-03 | CET | - | - | White | White or cream | Light brown | Bitter |
| BRS-CS01 | CET | PYT | - | White | White or cream | Light brown | Bitter |
| BRS-Dourada | CET | PYT | - | Yellow | Pink | Light brown | Sweet |
| BRS-Formosa | CET | PYT | UYT | White | White or cream | Light brown | Bitter |
| BRS-Gema de Ovo | - | PYT | - | Cream | White or cream | Light brown | Sweet |
| BRS-Kiriris | CET | PYT | UYT | White | White or cream | Light brown | Intermedium |
| BRS-Mulatinha | CET | PYT | UYT | White | White or cream | Light brown | Bitter |
| BRS-Novo Horizonte | CET | PYT | UYT | White | White or cream | Yellow | Bitter |
| BRS-Poti Branca | CET | PYT | UYT | White | White or cream | Light brown | Bitter |
| Cigana Preta | CET | - | UYT | White | White or cream | Yellow | Bitter |
| Corrente | CET | PYT | UYT | White | White or cream | Light brown | Bitter |
| Eucalipto | CET | PYT | - | Cream | White or cream | Light brown | Sweet |
| Vassoura Preta | CET | PYT | UYT | White | White or cream | Light brown | Bitter |
